# Editorial: Community series in neurogastroenterology – Focus on the gut-brain axis, volume II

**DOI:** 10.3389/fpsyt.2023.1339557

**Published:** 2024-01-11

**Authors:** Andreas Stengel, Yvette Taché

**Affiliations:** ^1^Department of Psychosomatic Medicine and Psychotherapy, University Hospital Tübingen, Tübingen, Germany; ^2^German Center for Mental Health (DZPG), Tübingen, Germany; ^3^Department for Psychosomatic Medicine, Charité Center for Internal Medicine and Dermatology, Charité-Universitätsmedizin Berlin, Corporate Member of Freie Universität Berlin, Berlin Institute of Health, Humboldt-Universität zu Berlin, Berlin, Germany; ^4^Vatche and Tamar Manoukian Digestive Diseases Division, Department of Medicine, University of California at Los Angeles (UCLA)/Digestive Diseases Research Core Center, UCLA David Geffen School of Medicine, Los Angeles, CA, United States; ^5^Veterans Administration (VA) Greater Los Angeles Healthcare System, Los Angeles, CA, United States

**Keywords:** doctor-patient communication, gut-brain axis, inflammatory bowel disease, nocebo, psychosomatic, sex hormones, stress

Following up on the previous Research Topic entitled “*Neurogastroenterology – Focus on the gut-brain axis*” published in 2021 (1) an update was planned this year reflecting the timeliness of the topic along with an increasing amount of data. The current *Community series in neurogastroenterology—Focus on the gut-brain axis—volume II* encompasses four articles, two original articles, one study protocol and one review.

Since inflammatory bowel disease seems to have a link to overlap with neurological and psychiatric symptoms/disorders, Liu et al. investigated possible alterations of the cerebral cortex in patients with inflammatory bowel disease in a large sample of 133,380 European subjects. While neither inflammatory bowel disease nor cytokines were associated with global cortical changes, the regional level cortical thickness and the surface area were altered and associated with the respective inflammatory bowel disease itself or cytokines such as interleukin-6 or the interleukin-6 receptor alpha (Liu et al.). These results, as pointed out by the authors, give rise to alterations of the gut-brain axis in this disease. Whether these results will have therapeutic (e.g., for monitoring of progression of the disease) implications will have to be further investigated.

A good doctor-patient communication is essential for the relationship and later for medical outcome, not only, but perhaps especially in patients with functional gastrointestinal disorders. Goebel-Stengel et al. investigated more than 5000 physician-patient conversations from more than 500 physicians. While physicians mostly assumed stress-related burdens as contributing factor, patients more often suspected food as a cause/trigger for their complaints (Goebel-Stengel et al.). Interestingly, despite the fact that physicians overall rated themselves as confident even in difficult patient conversations, doctors at the same time rated the physician-patient relationship just below the threshold for difficult interactions using a validated questionnaire. This likely gives rise to the need of (further) training of physicians in doctor-patient communication especially for difficult conversations e.g., with patients with functional gastrointestinal disorders.

One important component contributing to the pathogenesis of functional gastrointestinal disorders, also known as disorders of gut-brain interactions, is visceral (hyper)sensitivity. Labrenz et al. discuss in their review the interplay with stress in the modulation of visceral pain and the role of sex hormones, likely contributing to the higher prevalence of functional gastrointestinal disorders in women compared to men. The authors also critically highlight gaps in knowledge and propose the need for investigations across the lifespan to shed more light on this topic and for the development of personalized treatment strategies.

Lastly, a study protocol by Aulenkamp et al. delineates a randomized controlled trial investigating nocebo effects in healthy volunteers. While the placebo effect has attracted a lot of attention during the past four decades, the nocebo effect, related to negative expectation on treatment outcome, has been less investigated and only during the past 15 years. In this study, a multiple-threat paradigm as well as rectal distensions and cutaneous thermal stimuli will be used to investigate the effect of negative expectations on visceral and somatic pain (Aulenkamp et al.). This protocol will not only be assessing cross-sectionally but also the persistence of effects over time and combine psychophysiological and neuroendocrine parameters. The enhanced understanding of the role of nocebo effects in patients with disorders of gut-brain interactions will help to better design clinical studies for these patients, taking into account the nocebo effect(s) and ultimately the impact on treatment options.

Collectively, this community series on the gut-brain axis stretches the arc from inflammatory bowel disease to functional gastrointestinal disorders with all studies ultimately thriving to improve medical care for patients with disorders of gut-brain interactions.

## Author contributions

AS: Writing – original draft, Writing – review & editing. YT: Writing – review & editing.
